# Classroom Enjoyment: Relations With EFL Students’ Disengagement and Burnout

**DOI:** 10.3389/fpsyg.2021.824443

**Published:** 2022-01-13

**Authors:** Haoting Li

**Affiliations:** Marxist Institute, Heilongjiang University, Harbin, China

**Keywords:** classroom enjoyment, EFL students’ burnout, EFL students’ disengagement, language education, engagement

## Abstract

As learner burnout and disengagement affect the functioning and performance of college learners and can also influence future career-related well-being, they can be an issue for higher education organizations. Conversely, the discipline of language education has experienced an emotional turn, primarily triggered by Positive Psychology, and the scholars and students have been affected by various emotions. One of the seldom mentioned constructive emotions concerning learners’ disengagement and burnout is enjoyment in learning a foreign language, as has been demonstrated by literature reviews. It is important to note that this review helps scholastic institutions and policymakers in the scholastic community to take into consideration the role of constructive emotions, specifically enjoyment, and their constructive influence on language education in diminishing learners’ challenges in the learning process.

## Introduction

Engagement is an important notion in teaching today and is important for all learners (Hiver et al., 2021); learning cannot take place without engagement. Highly engaged learners perform better while less engaged learners perform poorly, are more absent, and are at risk of eventually dropping out of school (Lei et al., 2018). Learner engagement is conventionally represented as a three-dimensional concept of behavioral, emotional, and intellectual dimensions that are interrelated, supportive, yet distinct (Chang et al., 2016). Engagement and disengagement could be conceptualized as parallel, but well-separated multi-layered concepts with behavioral, intellectual, emotional, and social aspects (Bergdahl et al., 2019). Disengagement has been established as a corresponding concept to engagement and thus, may be conceptualized in ways other than just lack of engagement, like absenteeism (Salmela-Aro et al., 2017). According to studies, disengagement may be associated with various types of induction circumstances, including the inadequate design of educational activities (Bergdahl et al., 2019). On the one hand, learners’ engagement in school is related to grades and retention frequencies; on the other hand, investigations have demonstrated that disengaged learners are lagging and the early signs of disengagement must be determined to attend to and to avoid a downward spiral (Wang and Eccles, 2013). Furthermore, learners who left high school had high unemployment and imprisonment rates, which costs high for people, families, and society. Thus, some scholars suggest that the fight against disengagement is related to national well-being (Vallée and Ruglis, 2017).

Besides, students’ disengagement, as one of the broadest issues in teaching, burnout prevents learners from attaining their scholastic goals. Learners experience burnout when they overwork for a long time when they have no control over the circumstance, when they have little or no zeal for work, or when they have no reason to move on (Moghadam et al., 2020). Indeed, burnout is characterized as a mental syndrome defined by gradual emotional malaise, loss of inspiration, and decreased excitement (Vizoso et al., 2019). Learners experience the emotional disorder, an inclination toward depersonalization, and a decline in the individual performance called scholastic burnout in the educational cycle, due to course stress, course load, or other mental elements (Parker and Salmela-Aro, 2011). Even though learners are not employees, according to the viewpoint of psychology, their studies include organized activities like class attendance, classwork submission, which may be regarded as “work” (Lin and Huang, 2014). Burnout can lessen instructive engagement levels, such as class participation, plan of a school assignment, and following teachers’ directions (Morgado et al., 2017). In academic studies, while there exists a lot of literature on educator burnout, learner burnout has been less of a concern. It has nonetheless become evident that burnout can occur not only in educators, but also in learners (Robins et al., 2018; Karimi and Fallah, 2021).

Alternatively, foreign language learning development significantly centers on emotions. Short-termed, emotional, expressive physical reactions that assist with adjusting to the difficulties faced during significant life occurrences are known as emotions (Reeve et al., 2019). Positive psychology attempts to find ways to tackle negative emotions, supporting the expansion of ways to construct positive emotions, nurture more engagement, and increase the gratitude for meaning in life (MacIntyre et al., 2019). Negative emotions are linked with certain behaviors like anger, concern, or the need to escape and get rid of disgust while constructive emotions in language education are more than just satisfying emotions. Students with constructive emotions will be more aware of things in the classroom, more conscious of language input, and will be able to absorb more of the foreign language. One of these constructive emotions that are gaining more and more attention in language learning is called enjoyment, which is conceived as a condition of encountering pleasure in L2 education (MacIntyre et al., 2019; Wang et al., 2021). Enjoyment is one form of constructive feeling that has attained the most interest ever since the beginning of the positive psychology movement and it is one of the most commonly encountered constructive emotions (Dewaele and Li, 2020). Reviews of the qualitative literature on the educational setting had paid more attention to constructive emotions rather than negative ones (Zhang et al., 2020). Indeed, to the best of the researcher’s knowledge, very few inquiries (Li, 2020) so far have been done to scrutinize the occurrence of negative emotions such as burnout among language learners. Considering that emotions especially constructive types may have diverse shapes and theoretical configurations in various settings (Li et al., 2018; Wang and Guan, 2020), and the mediating effect of classroom enjoyment in language learning (Zhang et al., 2020), the present review intends to focus on classroom enjoyment and conceptualize its role on student burnout and engagement in the English as a Foreign Language (EFL) context, and explore how it grows in an EFL context to alleviate learners’ problems in the process of learning.

## Review of Literature

### Burnout

The notion of burnout was originally introduced in the human services industry, where people do professional work but consequently sustained to other professions and working collections (Maroco and Campos, 2012). Burnout has been defined by three elements, namely exhaustion, measured using items related to fatigue, despite the cause of these emotions, indifference to activity, or cynicism, associated with aloof attitudes, and finally, diminished professional effectiveness due to social and non-social dimensions of professional (or scholastic) performance (Maslach et al., 2001). The central element of scholastic burnout is emotional fatigue as a result of long-term scholastic stress. It denotes the sense of being overburdened and exhausted of resources (Kim et al., 2014). A deconstructive, indifferent, ruthless, or distant attitude with regards to one’s job or those involved is known as cynicism. Ineffectiveness alludes to the inclination of feeling incompetent or poorly performing one’s job (Robins et al., 2018). Cynicism and ineffectiveness heighten with emotional malaise as learners encounter an absence of progress over time (Parker and Salmela-Aro, 2011).

### Classroom Enjoyment

Classroom enjoyment is a high activation constructive attainment emotion that results from continuous educational activities or assignments that have a beneficial effect on different L2 educational results, such as L2 inspiration, engagement, and educational attainment (Dewaele and Li, 2020; Li, 2020; Guo, 2021). Students, who feel enjoyment, manage the attaining activities in which they are involved and/or recognize the consequences of the activities as individually meaningful (Pekrun et al., 2007). In this sense, educational enjoyment can be discerned as the joy of learning when the student feels that he/she can value the content and manage and complete the activities he/she is encountering. For this reason, enjoyment is considered as crucial for later satisfaction, which complements scholastic success (Ainley and Hidi, 2014).

### Disengagement as a Multidimensional Construct

Disengagement is defined by low energy, decreased participation, and ineffectiveness (Maslach et al., 2001). The tension and malaise that result from encountering over-demanding work are referred to as low energy (Schaufeli et al., 2002; Han, 2021). Disengagement alludes to learners’ passiveness to assignments and activities, including leaving one’s work and encountering deconstructive emotions about the work overall (Fredricks et al., 2004). Decreased participation, however, is the feeling that one has lost interest in one’s job and its meaning. Ineffectiveness is defined by a sense of incompetence when performing one’s job (Schaufeli et al., 2002). The three dimensions constituting engagement are behavioral, emotional, and intellectual as they also apply to disengagement, implying a multi-faceted concept (Wang and Eccles, 2013). Brint and Cantwell (2014) suggested five aspects of disengagement based on learners’ values, inspiration, learning behaviors, academic association, and competitive involvement, where disengagement could take place in one or more aspects. Disengagement takes place when learners do not value learning, when education has a low priority, and when the study is viewed as solely obtaining a degree. Motivation disengagement takes place when learners are discouraged to attain their learning objectives, whereas behavioral disengagement is witnessed due to limited learning time, failure to attend classes, and failure to complete tasks. Association disengagement takes place when learners do not associate with educators and peers, and competitive participations are non-scholastic activities, ranging from leisure and social activities to paid employment (Brint and Cantwell, 2014).

## Implications and Future Directions

The present review indicated that learners who undergo burnout act unwell in the classroom since they feel fatigued, bored, and unsatisfied about tasks and accountabilities so their involvement in language learning declines. This is due to their dearth of energy and the internal assets required enduring the expected academic pressures. Moreover, when learners are labeled as disengaged, or burned out, it indicates that classroom practices do not activate their attentiveness. Therefore, prompting classroom enjoyment can improve their engagement. In the context of L2, enjoyment can be seen as a constructive, educational-promoting attainment emotion associated with increased engagement (Botes et al., 2021).

The current review offers central implications for the vitality of EFL courses. The students can take advantage of the point that enjoyment has deemed a contrivance that stimulated their attentiveness and assisted them in the learning engagement so they become more interested and involved in their learning procedure and experiencing enjoyment is recognized as a verified manner to form a communally related learning environment between teachers and learners and even their peers that all help them to overcome their burnout (Jiang and Dewaele, 2019). If teachers desire to plan some chances for learners’ enjoyment to acquire a foreign language, they should be supposed to lessen apprehension. Enjoyment inspires students to attend more in the classroom, as it considers their resilience that helps them to overcome the symptoms of burnout in case of challenges. Enjoyment in language education is maintained to assist language students with better focusing on, processing, and mastering their target language, and concluding from the literature reviews above, learners’ disengagement appears to be a recurring problem in learning that can adversely affect scholastic results (Fredricks et al., 2004). Disengaged students tend to alienate themselves in the class by making no effort, giving up easily when encountering difficulties, and moving away from educational activities (Reeve et al., 2019). Subsequently, curriculum designers should strive to develop some approaches or strategies in the language environment and develop assignments that assist language students with increasing their enjoyment. In addition, the results of this review increase educators’ consciousness of the procedures that they can execute to promote enjoyment in associations with their students in a foreign language class. Educators must be fully aware of the signals they could utilize in the development of enjoyment in the classroom.

Furthermore, this review is noteworthy for educators as they can have a basic role in generating a constructive classroom atmosphere among EFL learners and as the classroom enjoyment should be provoked by educators, they should concentrate on the enhancement of their instructional abilities and try to develop learners’ syllabus by cultivating the classroom setting and incorporating instruction with enjoyment (Guo, 2021). In the same vein, learners can be more engaged in the class when educators offer more scholastic and emotional support, show interest and passion for instructing, and seek to build warm, personal, and compassionate connections with them, so enjoyment has thus been hypothesized as a central component of learners’ engagement. When students enjoy learning, their inclination to withstand their learning determinations may be preserved straightforwardly. In a nutshell, more empirical studies should be conducted to consider both the predictor and mediator function of enjoyment in the language learning context.

## Author Contributions

The author confirms being the sole contributor of this work and has approved it for publication.

## Conflict of Interest

The author declares that the research was conducted in the absence of any commercial or financial relationships that could be construed as a potential conflict of interest.

## Publisher’s Note

All claims expressed in this article are solely those of the authors and do not necessarily represent those of their affiliated organizations, or those of the publisher, the editors and the reviewers. Any product that may be evaluated in this article, or claim that may be made by its manufacturer, is not guaranteed or endorsed by the publisher.
